# What Can We Learn from Synaptic Connectivity Maps about Cerebellar Internal Models?

**DOI:** 10.1007/s12311-022-01392-6

**Published:** 2022-04-07

**Authors:** Ludovic Spaeth, Philippe Isope

**Affiliations:** 1 Institut des Neurosciences Cellulaires et Intégratives, CNRS, Université de Strasbourg, 67084 Strasbourg, France; 2 Present Address: Dominick P Purpura Department of Neuroscience, Albert Einstein College of Medicine, Bronx, NY USA

**Keywords:** cerebellum, synaptic transmission, sensorimotor adaptation, internal models

## Abstract

The cerebellum is classically associated with fine motor control, motor learning, and timing of actions. However, while its anatomy is well described and many synaptic plasticity have been identified, the computation performed by the cerebellar cortex is still debated. We, here, review recent advances on how the description of the functional synaptic connectivity between granule cells and Purkinje cells support the hypothesis that the cerebellum stores internal models of the body coordinates. We propose that internal models are specific of the task and of the locomotor context of each individual.

## Introduction

One of the major role of the cerebellum in sensorimotor adaptation is to learn to predict the future in order to adjust or correct the course of our actions [1–3]. To do this, many studies demonstrated that the cerebellum stores internal models of the motor apparatus, which combine motor commands with the current body state in order to predict an expected sensory feedback [4–7]. The comparison between the actual and the expected sensory feedback defines a prediction error that allows for correction, re-adjustment of the internal models, and ultimately promotion of behavioral adaptation. At the microcircuit level, the adapted behavior would correspond to modified network dynamics underpinned by a re-organization of the excitatory and inhibitory synaptic inputs at different cerebellar connections. Synergies of synaptic plasticity can ensure re-adaptation to a constantly changing environment. Therefore, the description of the functional synaptic organization (i.e., the connectivity maps)under normal or perturbed conditions should help understand how cerebellar internal models enable sensorimotor adaptation. After introducing the topography of cerebellar climbing and mossy fiber inputs, we will discuss how connectivity maps between granule cells (GC) and Purkinje cells (PC) can shed light on internal models implementation in the cerebellum.

## Climbing fibers define sagittal functional microzones

**Fig. 1:**
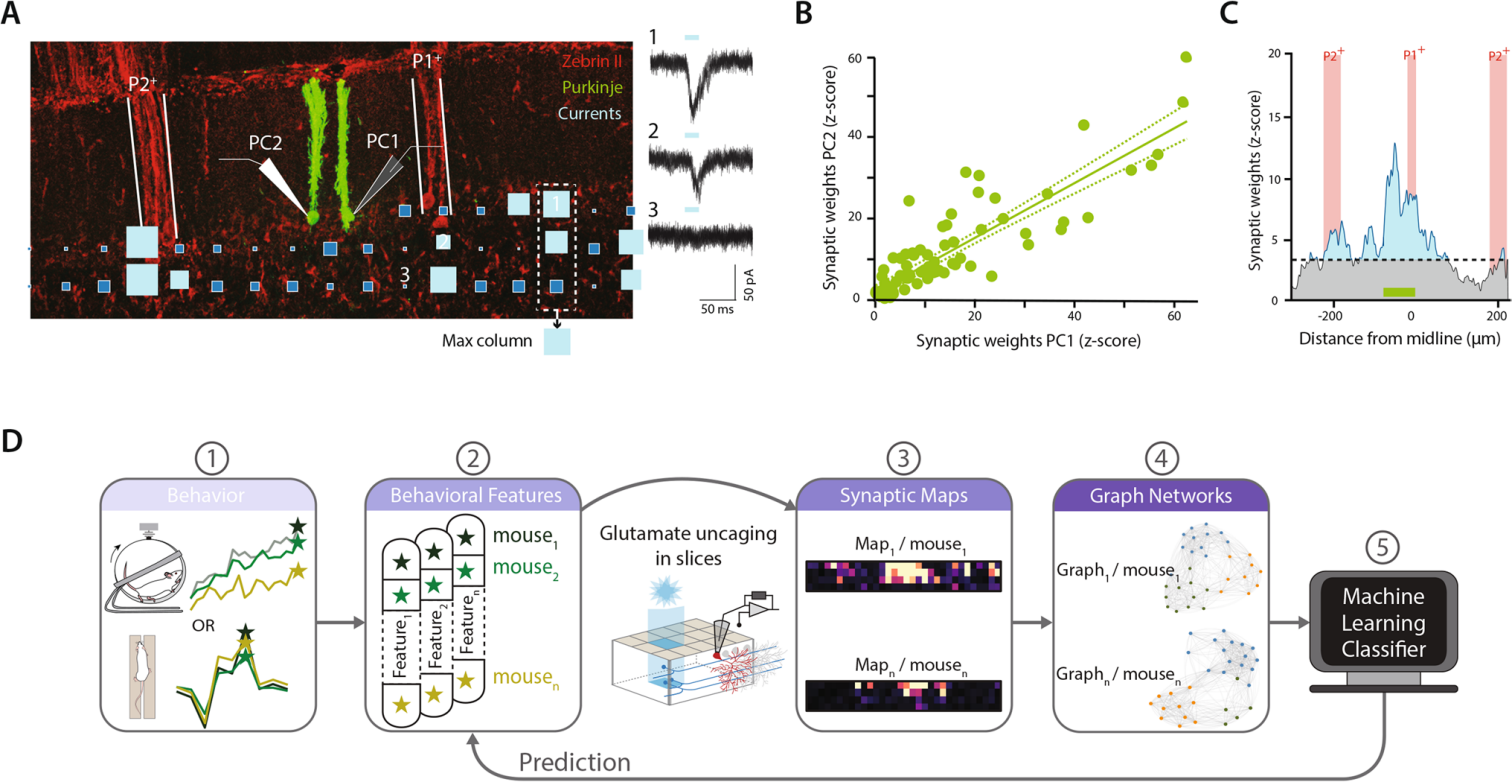
**GC-PC connectivity maps. A**, left. PCs (green) are recorded using patch-clamp and filled with biocytin for reconstruction and positioning (zebrin bands labeled in red). RuBi-Glutamate is uncaged using patterned blue light illumination, while GC-PC postsynaptic currents are recorded. The maximum of each GC column is used to determine a 1D pattern of connectivity for each PC (see C). Note the patchy organization of connected sites.  *Right*, examples of excitatory postsynaptic currents evoked by glutamate photolysis in corresponding sites. **B**, correlation between postsynaptic currents recorded in neighboring PCs indicating that they have similar connectivity maps. **C**, median of the 1D connectivity maps (z- scored) across animals (n = 18), illustrating that some specific GC layer columns are connected to midline PCs in many animals. Z-score < 3 corresponds to silent sites (i.e., illuminated GC layer of 41 × 41 x ~ 100 µm eliciting current < 15 pA in the recorded PC). Note local and distal hotspots of GC-PC connections. A, B and C Adapted from [23]. **D**, Strategy to map synaptic connections in neural networks and correlate it to animal behavior. Panel 1, animals training in different locomotor contexts. Panel 2, extraction of behavioral features related to sensorimotor adaptation for each mouse (e.g., distance traveled in a wheel or gait symmetry in a corridor). Panel 3, GC-PC synaptic connectivity maps description in a specific area of the cerebellum involved in the behavior. Panel 4, determination of the structure of these maps using mathematical algorithms from graph theory. Panel 5, the structure of the maps and the behavioral parameters of each mouse are used to build a classifier that can predict animal behavior from the synaptic map. The classifier is validated by testing its efficiency on a different set of data. Adapted from [24]

The cerebellar cortex receives two major excitatory inputs, the climbing (CF) and the mossy fibers (MF), which are both topographically organized [8–11]. A given group of neighboring PCs is contacted by CFs originating in a discrete region of the inferior olive and conveying information from the same receptive fields, defining a functional unit called microzone [12–15]. Since olivary cells are coupled by gap junctions and fire together, this pathway leads to synchronized activation of PCs from the same microzone [16]. Microzones were therefore defined as parasagittal zones of the cerebellar cortex composed of a group of PCs, the molecular layer interneurons (MLI) in the vicinity and the GCs located underneath  [8, 9, 17]. Since PCs project to a specific group of nuclear neurons from which a subgroup project back to olivary cells, microzones constitute the cortical part of an olivo-cerebellar module processing information related to a defined area of the body [8]. Microzone boundaries can be identified by a family of neurochemical markers, which are specifically expressed in sagittal bands of PCs [15, 17] (Fig. 1A). When these markers are labeled, the cerebellar cortex looks like a zebra displaying positive and negative bands; hence, they are collectively called zebrins [17]. Many studies have also demonstrated that microzones have anatomical and functional regional differences leading to a large diversity of information processing in individual microzones [18–22]. Therefore, an exhaustive functional description of the synaptic communication between microzones is necessary to understand how they collectively influence motor coordination.

## The mossy fiber to Purkinje cell pathway: an anatomy favoring internal model implementation

As opposed to the strict sagittal orientation of the CF inputs, MF inputs, which contact GCs, project to many different GC layer areas in a given lobule sending the same information into multiple microzones [11, 25]. Indeed, in vivo electrophysiological micro-mappings of the GC layer demonstrated that MF receptive fields in the GC layer have a fractured and redundant somatotopy [26]. Furthermore, many pre-cerebellar nuclei carrying distinct modalities project MFs to the GC layer of the same microzone 27, 11. In the anterior lobe, for example, lobule IV and V receive MFs from the spinocerebellar tract (e.g., DSCT, dorsal spinocerebellar tract) and the external cuneate conveying proprioceptive information from the hindlimbs and the forelimbs, respectively, as well as from the pontine nuclei, which carry information from the cerebral cortex such as motor plans and motor commands [28]. Therefore, individual GCs can integrate multimodal information [26, 29] but see [30] that combine both motor plans or commands and sensory feedback. GC axons, the parallel fibers (PF), which travel across multiple microzones, make excitatory synapses onto both local and distant PCs and molecular interneurons (MLI) located at millimeters from the incoming microzone [31, 32]. Within a given lobule, PFs likely control the PC population code via direct excitation and indirect inhibition in a wide array of microzones.[33–35]. Moreover, many experiments demonstrated that the GC-PC connection is a major site of information storage in the cerebellar cortex through CF-dependent plasticity: GC-PC synapses are depressed when CF and PFs are active in conjunction and potentiated when only PFs are activated [36, 37].

The organization of MF inputs into the GC layer expands encoding possibilities and is in agreement with the long-standing Marr–Albus–Ito hypothesis, suggesting that expansion recoding in the GC layer enables pattern separation of similar MF inputs [38–42]. Moreover, the convergence of sensorimotor inputs from the spinal cord and cortico-pontine inputs suggests that the GC layer can compute motor commands as well as sensory context or feedback, a prerequisite configuration for the implementation of forward internal models. Indeed, in vivo PC recordings during movement have shown that modulation of PC discharge can lead or lag movement onset [2, 43, 44]. Recent experiments have demonstrated that in a operant task in which monkeys have to follow a cursor on a screen PC discharge can predict the position error of the hand by a few hundred milliseconds ahead and compute in return the visual feedback shortly after the movement [6] (see also [3, 45]). Since the modulation of PC discharge relies on the combined activity of GCs and MLIs, these results suggest that the cerebellar cortex stores an internal model of this skilled movement. In a different task, in which mice move a handle to get a reward, GCs encode a predicted reward, suggesting that internal models are not only sensorimotor engrams [46]. Therefore, understanding how the cerebellar cortex encode sensorimotor information and store internal models requires determining how the GC-(MLI)-PC pathway is functionally organized.

## Interrogating the spatial organization of the GC-PC connection

Glutamate uncaging combined with patch-clamp recordings can be used on acute brain slices [47–49]. This approach allows the systematic excitation of single or small groups of presynaptic cells [50, 51] while monitoring the resulting synaptic inputs in the target cell soma (Fig. 1A). Uncaged glutamate excites mostly dendritic or somatic receptors preventing the direct excitation of neighboring axons as opposed to electrical or optogenetic stimulation. When glutamate uncaging is combined with a system enabling specific and localized restricted illumination (laser scanning or patterned light illumination), the spatial organization of the presynaptic cells as well as their synaptic weights on the target cell can be reconstructed [23, 24, 52, 53] (Fig. 1A, B). Hence, each recording yields a single connectivity map. This method is particularly well adapted to the study of the GC-PC connectivity maps as PCs from identified microzones can be targeted using transgenic mice expressing fluorescent zebrin markers [54]. Therefore, the use of caged-glutamate photostimulation and whole-cell patch clamp of PCs in identified zebrin bands allows the description of specific connectivity maps across different animals and eventually different behavioral conditions [23, 24].

## Spatially organized GC-PC connectivity maps as an implementation of internal models

Many in vitro and in vivo experiments demonstrated that while direct stimulation of PF beams can activate PCs along the beam, GC or MF activation actually modulate specific groups of PCs [55–58]. In vivo recordings of tactile receptive fields showed that in a given microzone GC layer, Golgi cells and MLIs are excited by the same area of the body, suggesting that incoming MF inputs lead to the activation of most cell types [14, 56, 59]. Conversely, PCs are excited by specific MF inputs targeting GCs in distant microzones, illustrating a functional selection of GC-PC synapses [23, 60]. Indeed, in two seminal studies, Jorntell and Ekerot [59, 61] demonstrated in vivo that PCs sharing the same PF beam are excited by different and specific receptive fields, but upon repeated high frequency PF stimulation PCs respond to all. Altogether, these results suggest the existence of a priori nonfunctional—or silent—GC-PC synapses (e.g., depressed by the CF-dependent LTD) that are potentiated—or awakened—via activity-dependent plasticity. This hypothesis was confirmed in vitro, using paired recordings and photostimulation studies in acute slices, showing that while PCs are always connected to local (i.e., underneath) GCs, a selection of distant hotspots of connected GC patches is interleaved with GCs making silent or nonfunctional synapses [23, 24, 62] (Fig. 1A, C). Silent synapses were also recently observed during development and in adult rodents [24, 63].

By repeating photostimulation and establishing excitatory GC-PC and GC-MLI connectivity maps in an identified set of microzones across many mice, several other operational rules were described [23, 24]: (1) neighboring PCs have a similar patchy connectivity map defining clusters of PCs spatially organized (Fig. 1A, B). While PC clusters are related to zebrin band, they overlap with zebrin boundaries, suggesting that a finer functional organization may coexist with zebrin band topography [17]. (2) MLIs from an identified microzone have different connectivity maps than local PCs, but they are similarly organized with local and distal patches of connected GCs. (3) Although functional connectivity is highly variable between animals, some specific areas of the synaptic maps are conserved between individuals, highlighting specific association between microzones through GC-PC synapses (Fig. 1C). (4) Connectivity maps are plastic and can be modified by protocols of PF stimulation, which lead to a reorganization of the map with potentiated or depressed GC sites, suggesting that they are sensitive to activity of the MF-GC-PC network. GC-PC maps were also recorded after mice underwent locomotor adaptation in different locomotor context [24]: a transient locomotor impairment (i.e., recovery following unilateral alteration of the sciatic nerve) or a training in a wheel for three weeks. These specific locomotor contexts induced both a sustainable increase in overall synaptic weights of GC-PC connections synapses and a specific reorganization of the connectivity maps. Moreover, using mathematical algorithms based on graph theory, we demonstrated that a synaptic connectivity map from a given mouse is correlated with the unique behavioral performance of this mouse in a simple task such as walking on a corridor or running in a wheel (Fig. 1D). These results suggest that GC-PC connectivity maps are specific engrams (i.e., internal models) of behavioral adaptation, allowing encoding of movement features in a context-specific manner throughout individual motor learning.

## Patchy organization of GC-PC maps and “sparse” coding in the cerebellar cortex

Altogether, these operational rules underlying microzonal communication in the cerebellar cortex are likely at the core of cerebellar computation [8]. The description of synaptic connectivity maps in cerebellar lobules could shed light on the combination of microzones that control a given adapted behavior. We postulate that local MF inputs in recipient microzones set a minimal framework of activated PCs while the patchy organization of GC-PC synaptic maps allows the spatial and temporal combination of an extended array of microzones by PFs. Such organization may reconcile Marr–Albus–Ito [38] and adaptive filter [39, 64] theoretical models with the recent studies describing GC activity using in vivo two-photon imaging [44, 65, 66]. In these theoretical models, a sparse coding strategy at the MF-GC synapses is a prerequisite for the expansion-recoding hypothesis and few GCs should be activated by a specific combination of MF inputs.

This assumption might appear in contradiction with the dense activation of GCs observed using two-photon imaging of genetically encoded calcium indicators in behaving mice or zebrafishes [44, 65, 66]. They demonstrated that large areas of the GC layer seem simultaneously active at specific moment of the task and correlated with sensorimotor or predictive reward inputs. Anatomical, physiological, and technical issues may explain this apparent discrepancy. MFs carrying information from a given body part target specific areas of the GC layer making many rosettes in a restricted area. This arrangement favors the aforementioned patchy organization of the GC-PC functional connectivity and may explain why two-photon imaging identified patches of dense GC activity. Nevertheless, since the majority of GC-PC synapses are silent [23, 24, 62], GC-PC connectivity can still be sparse. Moreover, short-term plasticity at the MF-GC synapses expands temporal integration in GCs from tens to hundreds of milliseconds [67], suggesting that in a given GC patch targeted by a combination of multimodal MFs, GC discharges may be split into a wide range of temporal patterns yielding GC population decorrelation. As explained in [40], this mechanisms may enable pattern separation even if GCs are densely activated. In two-photon GCamP6 imaging experiments, these patterns cannot be temporally discriminated and are merged into a single giant patch of simultaneously activated GCs. Heterogeneity of short-term plasticity was also described recently at the GC-MLI and GC-MLI-PC connection [33, 68], indicating that the temporal expansion performed by the GC layer is amplified in the molecular layer. Therefore, the spatial organization of the MF-GC-PC connections combined with the spread in the temporal integration window enables pattern discrimination and PCs to encode thousands of different patterns of information as suggested in the Marr–Albus–Ito hypothesis [69].

To conclude, patchy connectivity maps at the GC-MLI-PC connections may underlie the communication between specific microzones in the cerebellar cortex. Graph network properties of GC-PC connectivity maps suggest that context-dependent and task-specific internal models are encoded at the GC-PC synapses. Next step will be to describe how the feedforward inhibition influences connectivity maps and intermodular communication. Unraveling the rules governing the coordination between microzones will shed light on how the cerebellar cortex controls the output stage of the cerebellum. As an appealing perspective, it will be important to describe how the MF-GC-MLI pathway correlates microzones using genetically encoded voltage sensitive indicators combined with two-photon imaging. Knowing the combination of microzones and their sequence of activation in a specific behavior will help designing and refining cerebellar stimulation protocols to compensate for cerebellar dysfunction in pathological models [70].
